# Intra-aortic balloon pump experience: a single center study comparing with and without sheath insertion

**DOI:** 10.15171/jcvtr.2018.23

**Published:** 2018-09-24

**Authors:** Yücel Özen, Mehmet Aksut, Davut Cekmecelioglu, Mehmet Dedemoglu, Ozge Altas, Sabit Sarikaya, Murat Bulent Rabus, Kaan Kirali

**Affiliations:** Kartal Kosuyolu Heart Training and Research Hospital, Department of Cardiovascular Surgery, Istanbul, Turkey

**Keywords:** Intra-aortic Balloon Pump, Mechanical Support, Sheath Usage, Vascular Access

## Abstract

***Introduction:*** The mechanical circulation support used in treatment of low cardiac output at most is the intra-aortic balloon pump (IABP). Its usage fields are the complications occurring due to ischemic heart disease, disrupted left ventricle function, and the low cardiac output syndrome occurring during coronary artery by-pass surgery.

***Methods:*** During 28 years from 1985 to 2013, IABP support has been implemented to 3135 patients in our cardiac surgery operating theater and intensive care unit. The mean age of the patients was 61.4 ± 13.2 years (16-82). 2506 patients (80%) were the ones whom the cardiac surgery has been implemented. IABP support has been provided for 629 (20%) patients for medical treatment. We utilized IABP most frequently in coronary artery patients (70%). The first choice for placing the balloon catheter is the femoral artery in 3093 cases (98.7%).

***Results:*** The most frequently observed balloon complication was the lower extremity ischemia in 383 cases (12.2%).The leg ischemia was statistically significantly more frequent in patients with sheath (P=0.004). The extremity ischemia has developed in 4 of 12 patients with balloon placed from upper extremity. The local bleeding and balloon rupture were more frequent in patients whom the balloon has been placed without sheath. The mortality due to IABP has occurred in only 5 patients.

***Conclusion:*** Despite increase in IABP usage frequency rapidly, the complications due to catheter are still seen. We believe that the leg ischemia that is the most frequently seen complication can be prevented via IABP use without sheath.

## Introduction

Intra-aortic balloon pump (IABP) is the mechanical support device which is the one most frequently used in low cardiac output where the medical treatment is unsuccessful.^1^ By decreasing the afterload and increasing the pressure in roots of aorta, IABP leads the coronary arterial perfusion to increase. Hence, the myocardial oxygen requirement decreases. Since its use by Kantrowitz et al^2^ in 1968, IABP has been widely used in treatment of low cardiac output in many health care centers. The most frequent usage fields are unstable angina, complications developing secondary to ischemic cardiac disease, insufficient left ventricle function, and the low cardiac output syndrome developing during coronary heart surgery. Our aim in this study is to compare the complications in patients to whom the IABP with and without sheath has been implemented in our clinic.

## Materials and Methods

Between the years of 1985 and 2013, IABP has been implemented on a total of 3135 patients in our clinic. The study data has been obtained by retrospectively scanning the intensive care and operating theater records. The patients to whom the surgery has been implemented were grouped into 3 groups by IABP placement times as preoperative, intraoperative, and postoperative. The patients whom the balloon has been placed in intensive care unit before taking into operation were clustered in preoperative group, the ones whom the balloon has been placed during operation were clustered in intraoperative group, and the patients whom the balloon has been placed in intensive care unit follow-up after the operation were clustered in postoperative. The patients where the trans-femoral path has been used were clustered into 2 groups as use with and without sheath. These groups were compared in terms of developed complications. The patients where the IABP used with ECMO (extracorporeal membrane oxygenation) were excluded from the study.

### Statistical methods

Statistical analyses were carried out by utilizing the SPSS (Statistical Package for the Social Sciences) version 15.0 software. The definitive analyses were provided by using frequency tables of categorical variables. While comparing the intergroup variables, the “Pearson chi-square test” was utilized. *P* < 0.05 is accepted to be statistically significant.

## Results

The mean age of the patients was 61.4 ± 13.2 years (16-82), and 2880 (92%) were males. Most of these patients had (70%) coronary artery disease (CAD). The demographical data of the patients are presented in Table 1. Cardiac surgery was applied to 2506 (80%) of the patients. Isolated CABG was implemented on 1842 (58.7%) patients. The CABGs performed off-pump were included in this group. IABP support was provided for 629 (20%) patients for medical treatment purpose. Among these patients, 235 (7.5%) patients were the ones with cardiomyopathy waiting for heart transplantation. The IABP usage indications are presented in Table 2. IABP has been implemented on 372 patients (11.8%) preoperatively, on 1246 (39.7%) patients postoperatively. The most frequent balloon implementation point was femoral artery with 3093 cases (98.7%). The balloon has been placed in percutaneous path in 2670 (85.2%) patients, and surgically in 423 (13.5%) patients. In percutaneous implementation, the balloon has been placed by sending to femoral artery through sheath until the year 2000. In this period, the balloon was implemented without sheath in patients having thin femoral artery. Since this date, the sheathless balloon placement method is adopted as routine. The diameter of the used balloon catheter was 8.5F or 9.0F. In 42 (1.3%) patients where it has not been placed in trans-femoral method, the alternative methods have been preferred. IABP implementation points are given in Table 3.

**Table 1:** Demographic characteristics of patients

		**No.**	**%**
The mean age 61.4 ± 13.2 (16-82)	Male	2880	92
	Female	255	8
Diabetes mellitus		590	18.8
Hypertension		602	19.2
Chronic obstructive pulmonary disease		258	8.2
Hyperlipidemia		189	6
Smoking		1012	32.3
Peripheral artery disease		221	7
Coronary artery disease		2204	70.3
Rheumatic valvular disease		438	14
Ischemic valve disease		113	3.6

**Table 2:** Indications for use of IABP

		**No.**	**%**
**Surgery group**		2506	80
I- Preoperative application (372)	CAD	328	10.5
	Valve disease	34	1.1
	Combinated	10	0.3
II- Intraoperative application (888) (end of pump )	Isolated CABG	831	26.5
	Double procedures^a^	47	1.5
	Complex surgery^b^	10	0.3
III- Postoperative Application (1246)	Isolated CABG	1011	32.2
	Double procedures^a^	211	6.7
	Complex surgery^b^	24	0.8
**Medical group**	629	20	
Acute MI and complications, 304 (%)	1-Postinfarct VSD	81	2.6
	2-Ischemic mitral insufficiency	52	1.6
	3- Left ventricular dysfunction	150	4.8
	4- Arrhythmia	21	0.7
Unstable angina pectoris		33	1
LMCA		57	1.8
Cardiomyopathy		235	7.5

IABP: Inrtaaortic balloon pump, CAD: coronary artery disease, CABG: coronary artery bypass graft, MI: myocardial infarctus, VSD: ventricular septal defect, LMCA: left main coronary artery ^a^ The patients who applied 2 procedures with CABG either valvular or carotid surgery ^b^ The patients who applied 3 procedures as Bentall and CABG or CABG and double valve replacement.

**Table 3:** IABP application areas

			**No.**	**%**
Transfemoral, 3093 (98.7%)	Percutaneous 2670 (85.2%)	With sheath	934	29.8
		Without sheath	1736	55.4
	Surgical 423 (13.5%)	With sheath	160	5.1
		Without sheath	263	8.4
Alternative routes 42 (1.3%)	Transthoracic 30 (0.1%)	Direct	10	0.3
		Via graft	20	0.6
	Transaxillary 12 (0.4%)	Direct	8	0.2
		Via graft	4	0.1

IABP: Intraaortic aort balloon pump. ^a^ The total number of patients used IABP between 1985 and 2013: 3135

The most frequently seen complication in patients whom the balloon has been placed was lower extremity ischemia (383, 12.2%). Surgical intervention was implemented on 204 (6.5%) patients. The embolectomy has been implemented mostly (153, 4.9%). In 4 patients with advanced ischemia, the amputation was implemented. In 4 of 42 patients whose IABPs were placed in alternative paths, the upper extremity ischemia developed. In 2 of them, the surgical intervention was required. Also in 44 (1.4%) patients, there was bleeding in balloon placement points. Even though hospital mortality was 814 (25.9%), only 5 of them occurred as a result of complications due to balloon (Table 4).

**Table 4:** IABP complications

**Complications**				***No.***	***%***
**1-Extremity ischemia,** 387 (12.3%)	**Low extremity,** 379 (12%)	Medical		179	5.7
		Surgical 204 (6.5%)	Embolectomy	153	4.9
			Embolectomy + saphenous patch plasty	28	0.9
			Embolectomy + graft interposition	8	0.2
			Femoro-femoral bypass	2	0.06
			Fasciotomy	5	0.1
			Amputation	4	0.1
	**Upper extremity,** 4 (0.12%)	Medical		2	0.06
		Surgical 2 (0.06%)	Embolectomy	1	0.03
			Embolectomy + graft interposition	1	0.03
**2. Local bleeding**				44	1.4
**3. Hemolysis**				21	0.7
**4. Thrombocytopenia**				16	0.5
**5. Infections**				21	0.7
**6. Chronic serous discharge**				23	0.7
**7. Paraplegia**				5	0.1
**8. Aortic complications,** 7 (% 0.2)	**Dissection**			>3	>0.1
	**Rupture**			4	0.1
**9. Balloon Rupture**				82	2..6
**10. Cerebral Embolism**				3	0.1

IABP: Intraaortic aort balloon pump.

Paraplegia, which is a rare complication, developed in 5 patients. Thrombocytopenia developed in 16 patients, while hemolysis developed in 21 patients. In 7 patients, the aorta complication developed. Dissection occurred in 3 of these patients, and aorta rupture occurred in 4. And 4 of them were lost in early period. One of 3 patients where the cerebral emboli developed was lost in postoperative 35th day. The mortality due to balloon was found to be 0.16% with 5 patients (Table 4).

## Discussion

The need for increased use of IABP during cardiac surgery in the recent years has been reported by many groups.^3,4^ The first clinical application of IABP was in 1968 by Kantrowitz et al to support patients with cardiogenic shock due to myocardial infarction.^2^ The most frequently used one among the mechanical circulation support devices nowadays is the IABP. The most important reasons of that are its easy implementation, and its clear price advantage against other devices.^5^ IABP has two principal physiological effects; it improves coronary blood flow and myocardial oxygen supply by increasing diastolic perfusion pressure and reduces afterload by rapid balloon deflation in systole leading to reduced ventricular work and consequently decreased myocardial oxygen consumption.^6^ The real usage field of IABP, which is widely used in cardiology clinics and intensive care units, is the centers where the open cardiac surgery is applied. The aim of use in these centers can be divided into 3 as preventing the low cardiac output and severe myocardial ischemia in preoperative period, providing assistive support for patients who cannot leave the heart-lung device in intraoperative period, and preventing the low output in intensive care unit or medical-treatment-resistant arrhythmia in postoperative period.^7^ The most frequent balloon usage in patients whom we applied surgery in our clinic is in postoperative period with 1011 cases (32.2%).

Despite the positive hemodynamic effects of IABP, the complication rates related to IABPs are not low. In a study which reviewed the recent literature, the complication rates related to IABPs were reported to be between 32.6% and 50%.^8^ IABPs may lead to complications like thrombocytopenia, bleeding, injury to the aorta and iliac arteries, dissection, thromboembolism and leg ischemia. Thrombocytopenia and bleeding are the most commons complications.^9^ Additionally, complications related to malposition of the balloon catheter can be observed.^10^ Even though the incidence of arterial thrombosis and emboli development is decreased by implementing systemic heparinizing during IABP usage, the implementation’s effect increasing the bleeding in postoperative period may lead to stop or delay the usage. Among these complications, the most frequently observed one was the upper extremity ischemia with 387 cases (12.3%) (Table 4). The extremity ischemia was observed in lower extremity where the balloon catheter was placed and in patients whom the device was implemented with sheath. Comparing with patients without sheath, the result was found to be statistically significant (Table 5). The longer the IABP support continues, the more the ischemia development rate in that extremity increases. The reason of that ischemia which the balloon causes is the damage on vessel wall during placing the balloon catheter, the prevention of blood circulation by catheter, and vasospasm occurring due to low cardiac output. Female gender, advanced age, diabetes, and atherosclerotic heart disease are the factors speeding this complication up, and the development of lower extremity ischemia in such patients was found to be more frequent. In patients where the balloon catheter cannot be placed via femoral artery, the transthoracic, axillary or subclavian artery path is an alternative.^11^ In our clinic implementation, we placed IABP to 42 patients where the trans-femoral path could not be used (Table 2). Among these patients, upper extremity ischemia developed in 4 patients, and surgical intervention was required in 2 of them. In order to minimize the vascular complications in IABP implementation, we generally prefer sheathless balloon catheter implementation. The advantage of this method is to decrease the mechanical obstruction. But there may be the bleeding as severe leakage in insertion point of balloon catheter (Table 5). Local bleeding was found to be more frequent in sheathless group (*P* = 0.03). This bleeding can be taken under control easily via purse sutures around the catheter. Bleeding occurred in 44 of our patients at balloon insertion point. In 21 patients, the infection developed in insertion point of balloon. 18 of these patients were the ones where the balloon was installed surgically because it could not be installed in percutaneous path. Thrombocytopenia developed in 16 patients. In these patients, the thrombocytopenia cleared up after heparin implementation was cut, and balloon was removed. Balloon rupture developed in 82 patients. The balloon rupture was found to be statistically significantly more frequent in sheathless group (*P* = 0.02). As a result of increased quality of used material and the technological developments, the balloon rupture is observed less frequently in recent years. The rapid flow of blood into the balloon catheter or inability of performing filling must make us think balloon rupture. In that case, the balloon must be removed immediately. Cerebral emboli developed in 3 patients. The reason of the emboli may be removal of atherosclerotic plaque from aortic or carotid tips of balloon catheter during installing the balloon. Or the air emboli due to balloon rupture may have led that. For this reason, the length must be measure on the patient before installing the balloon, and the location of the balloon must be checked through transesophageal echocardiography or teleradiograph after the balloon is installed. One of the patients where cerebral emboli developed due to balloon rupture died on postoperative 43th day due to low cardiac output. Aorta rupture developed in 4 patients. By taking 2 of these patients under surgery, the rupture in aorta was restored with graft. But other 2 patients died. Aorta dissection developed in 3 patients. Two of them died. And 1 of the patients was not intervened. In control tomography, it was seen that the rupture has limited. It is very hard to predict this catastrophic situation. In cases where it is hard to push the guide wire forward during the process, we recommend to assess the abdominal aorta pathologies through abdominal ultrasonography.

**Table 5:** Hospital mortality

		**No.**	**Exitus**	**%**
Surgical group		2506	709	28.3
	Preoperative group	372	61	16.4
	Intraoperative group	888	220	24.8
	Postoperative group	1246	428	34.3
	Transfemoral access	3093	801	25.9
	Alternative access	42	13	30.1
Medical group		629	105	16.7
Balloon-related mortality		3135	5	0.16
Total hospital mortality		3135	814	25.9

Also the timing of IABP support is a controversial issue nowadays. The mortality is less than 20% in preoperative installation. But the mortalities in intraoperative and postoperative installations are almost 30% and 40%, respectively.^12^ Lavana et al evaluated the association between timing of IABP insertion and outcomes of the patients reporting mortality rates as 10% in preoperative group, 16% in the intraoperative group and 29% in the postoperative group. They concluded that preoperative IABP use was associated with reduction in hospital mortality.^13^ In study of Parissis et al, the preoperative mortality was found to be 18.2%, while the postoperative mortality was found to be 58.3%.^14^ Given results led to the suggestion of preoperative prophylactic IABP insertion in high risk patients before observing any hemodynamic compromise.^15^ In our study, the preoperative mortality was found to be 16.4%, while intraoperative mortality was found to be 24.8% and postoperative mortality was found to be 34.3%. Even though the total hospital mortality was 25.9%, the mortality due to balloon was found to be 0.16% (Table 6). The total hospital mortality in study carried out in our clinic at 1999 has been found to be 26.6%.^16^

**Table 6:** Complication comparison in transfemoral IABP applications

	**With sheath (n=1999)**		**Without sheath (n=1094)**		***P***
	**No.**	**%**	**No.**	**%**	
Extremity ischemia	282	14.1	101	9.2	0.
Amputation	4	0.2	0	0	0.04
Local bleeding	11	0.5	24	2.2	0.03
Local infection	9	0.4	9	0.8	1
Balloon rupture	26	1.3	42	3.8	0.02

IABP: Intraaortic aort balloon pump.

## Conclusion

The IABP of nowadays is a mechanical support device which is still used safely and widely in treatment of low cardiac output developing after cardiac surgery. The easiness of implementation of this mechanical support is the main reason of that it is first preference in all kinds of intensive care units and open heart surgery. As a result of our IABP implementations in single center, we think that the preoperative and early-intraoperative balloon implementations are safer than intraoperative and postoperative implementations. Even though the complications due to balloon catheter decreased in recent years, they still occur. We recommend sheathless usage in order to prevent the extremity ischemia which is the most frequent complication developing due to IABP.

## Limitations

This is a retrospective study, and not all the records of old patients could be reached. Since the medium- and long-term follow-ups of patients could not be reached, the long-term mortality rates could not be calculated.

## Ethical approval

The data have been gathered and analyzed retrospectively, after approval of local ethical committee.

## Competing interests

The authors declare that they have no competing interests.
